# Genomic surveillance of enterovirus D68 circulating in 2025 reveals the emergence of a novel A2/B3 recombinant lineage

**DOI:** 10.1128/spectrum.01005-26

**Published:** 2026-08-12

**Authors:** Amary Fall, C. Paul Morris, Omar Elgazayerly, Tianqi Wu, Omar Abdullah, Julie M. Norton, Andrew Pekosz, Eili Klein, Heba H. Mostafa

**Affiliations:** 1 Department of Pathology, Division of Medical Microbiology, Johns Hopkins School of Medicine 1500 , Baltimore, Maryland, USA; 2 Integrated Research Facility, Division of Intramural Research, National Institute of Allergy and Infectious Diseases, National Institutes of Health, Frederick, Maryland, USA; 3 Faculty of Medicine, Alexandria University 54562, Alexandria, Egypt; 4 W. Harry Feinstone Department of Molecular Microbiology and Immunology, The Johns Hopkins Bloomberg School of Public Health 25802 , Baltimore, Maryland, USA; 5 Department of Emergency Medicine, Johns Hopkins School of Medicine 1500 , Baltimore, Maryland, USA; 6 Center for Disease Dynamics, Economics, and Policy 439584 , Washington, DC, USA; The George Washington University School of Medicine and Health Sciences, Washington, DC, USA

**Keywords:** recombination, EV-D68, enterovirus

## Abstract

Enterovirus D68 (EV-D68) has re-emerged over the past decade as a significant respiratory pathogen associated with severe respiratory disease and acute flaccid myelitis. Its circulation has typically followed a biennial pattern, with predominance in late summer and early fall, a pattern that was temporarily disrupted during the COVID-19 pandemic. Surveillance in 2025 revealed off-season circulation of EV-D68. This study describes the genomic characteristics of the 2025 EV-D68 viruses and the clinical features of affected patients. Between May and December 2025, remnant respiratory specimens positive for rhinovirus/enterovirus were screened for EV-D68 and subjected to whole-genome sequencing. Phylogenetic analyses were performed using maximum-likelihood methods. Recombination was assessed using subgenomic phylogenies, SimPlot similarity and BootScan analyses, and read-level inspection. Among 1,321 patients tested, 147 (11.1%) were EV-D68-positive, and 119 (81.0%) yielded complete genomes. EV-D68 positivity increased in July 2025, peaked in August (~21%), and remained elevated through September and October, exceeding levels observed in 2024. Patients had a median age of 36 years, with infections disproportionately affecting older adults. Phylogenetic analysis demonstrated exclusive circulation of subclade A2. Five genomes formed a distinct recombinant lineage (A2-Re). Subgenomic phylogenies showed clustering with A2 viruses in the P1 region and with B3 viruses in the P2–P3 regions. SimPlot and BootScan analyses identified a recombination breakpoint near the 2A/2B junction (~nt 3,700). The recombinant lineage was associated with temporally clustered cases in September–October. These findings demonstrate recombination between distinct EV-D68 subclades and underscore the importance of whole-genome surveillance for accurate viral characterization. Continued genomic monitoring is essential for detecting emerging variants with potential implications for transmissibility, pathogenicity, and public health preparedness.

**IMPORTANCE:**

This study highlights an increased off-season circulation of Enterovirus D68 (EV-D68) and a higher burden of disease in adults in 2025. The identification of a novel A2–B3 recombinant lineage provides evidence of ongoing viral evolution through recombination, a mechanism that may alter transmissibility, virulence, or immune responses. Detection of this lineage in temporally clustered cases suggests local transmission and underscores the potential for rapid spread of newly emerged variants. These findings emphasize the limitations of partial genomic approaches and the critical role of whole-genome sequencing in accurately characterizing circulating strains and identifying recombination events. Enhanced genomic surveillance is essential to detect emerging variants in real time, inform diagnostic assay performance, and support public health responses. Continued monitoring of EV-D68 evolution will be important for anticipating changes in disease burden, guiding clinical awareness, and strengthening preparedness for future outbreaks.

## INTRODUCTION

Enterovirus D68 (EV-D68), a member of the *Picornaviridae* family, has re-emerged as a significant human pathogen over the past decade. EV-D68 primarily targets the respiratory tract, leading to disease manifestations from mild upper-respiratory infection to severe respiratory distress requiring hospitalization (1–3). Of public health concern is its established association with acute flaccid myelitis (AFM), a neurological disease characterized by acute limb weakness and paralysis, predominantly in children (3–6).

Since its widespread re-emergence in 2014, EV-D68 activity in the United States has followed a characteristic biennial pattern, with major epidemic peaks typically occurring during even years (1, 2, 5, 7–10). These outbreaks have typically demonstrated a late summer to early fall predominance (11). However, disruptions in respiratory virus circulation during the COVID-19 pandemic, along with ongoing viral genetic diversification, have raised important questions about whether this historically predictable seasonal and biennial pattern may be evolving. Notably, previous data from our group indicated that the biennial pattern was restored in 2022 and 2024 (10).

The evolution of EV-D68 has been marked by the global circulation of multiple genetic clades. Following the major 2014 outbreak driven by subclade B1, subsequent epidemic waves have shown diversification within clades A and B, with subclades B2 and B3 predominating in recent years (1, 5, 7–10). The diversification within different enterovirus species is driven by two principal evolutionary forces: the accumulation of point mutations and genetic recombination (12–16). Recombination is a well-recognized mechanism of viral evolution, allowing the exchange of genomic fragments between related viruses during co-infection of a host cell (17–19). Such events can give rise to novel lineages or subclades with altered pathogenicity, fitness, or antigenic characteristics, potentially posing challenges for both surveillance and outbreak control (20–23).

Although recombination is known to contribute to enterovirus evolution, only rare sporadic recombination events have been reported for EV-D68 (24–28). The identification and characterization of these recombinant genomes are critical for understanding the full scope of the virus’s evolutionary dynamics and adaptive potential.

In this study, we report an increased circulation of EV-D68 in Maryland in 2025 and describe the genomic identification and characterization of a novel recombinant EV-D68 strain.

## MATERIALS AND METHODS

### Study population and sample collection

Remnant clinical specimens were collected between May and December 2025 from patients receiving care within the Johns Hopkins Health System (JHHS), which serves inpatient and outpatient populations through five acute care hospitals and multiple ambulatory practices and covers a large geographical area in Maryland, Virginia, and DC. Respiratory samples that tested positive for rhinovirus/enterovirus (RV/EV) using the ePlex respiratory pathogen panels were collected for enterovirus screening and whole-genome sequencing (IRB00221396). Clinical and demographic data were collected as previously described (29) through a bulk query of the electronic medical record system.

### RNA extraction, EV-D68 screening, and whole-genome sequencing

Viral RNA extraction was performed with the Chemagic Viral DNA/RNA 300 Kit. Samples were screened for EV-D68 by real-time reverse transcription polymerase chain reaction as described previously (7, 30, 31). Whole-genome sequencing was performed as previously described (1, 10). Amplicons from two overlapping PCR reactions yielding fragments of approximately 4,380 and 3,200 bp were pooled and barcoded using the Native Barcoding Genomic DNA Kit (EXP-NBD196; Oxford Nanopore Technologies) according to the manufacturer’s instructions. Sequencing was conducted on a PromethION 2 Solo (P2) (Oxford Nanopore Technologies) using R10.4.1 flow cells. Raw FASTQ files were analyzed using a custom in-house pipeline. Primer sequences were trimmed with cutadapt (version 3.5) (32), and the closest reference genome was identified by BLASTn (33) against a curated Enterovirus database. Reads were aligned to the reference genome using minimap2 (version 2.30) (34), then sorted and indexed with Samtools (version 1.10) (35). Consensus genomes were generated and polished with Medaka (Version 2.1.1) (36). Variants were filtered with ARTIC tools using custom versions of artic_vcf_filter, artic_make_depth_mask, and artic_mask (37). Final consensus sequences were generated using bcftools (38).

### Phylogenetic and recombination analyses

Complete genomes were aligned with reference genomes from GenBank using Mafft (version 7.450). Maximum likelihood trees for complete genomes were carried out using IQ-TREE 3 (version 3.0.0), with 1,000 bootstrap replicates. The ModelFinder, implemented in IQ-TREE 3, was used to select the best-fit nucleotide substitution model.

Recombination detection was performed using multiple complementary approaches to ensure reliability. Whole-genome alignments of representative Enterovirus D68 strains were generated with MAFFT (v7). Phylogenetic incongruence between the P1, P2, and P3 regions was further assessed using maximum-likelihood trees constructed in IQ-TREE 3 with 1,000 bootstrap replicates. To visualize recombination breakpoints and confirm parental relationships, SimPlot (v3.5.1) was used to perform similarity and Bootscan analyses using a 200 bp sliding window and 20 bp step size. In addition, BAM alignment files were inspected in Integrative Genomics Viewer (IGV) (V2.19.6) to assess possible co-infections or mixed subclade signals within the samples analyzed. Sequencing of two samples was repeated to confirm the reproducibility of our results.

## RESULTS

### Demographic and clinical characteristics of EV-D68-positive patients

Among 1,321 patients tested for EV-D68, 147 (11.1%) were EV-D68-positive (Table 1). The overall cohort had an age range of 0 to 102 years, with a median age of 16 years, whereas EV-D68-positive patients ranged from 0 to 85 years, with a median age of 36 years. EV-D68 infections were observed across all age groups but showed a predominance of older age groups compared with EV-D68-negative patients (in individuals aged ≥65 years [21.1%] and 45–64 years [20.4%]).

**TABLE 1:** Demographics and clinical characteristics of patients infected with EV-D68 and other RV/EV at the Johns Hopkins Health System in 2025

	Positive EV-D68	Negative EV-D68	Total
Total	147 (11.1)	1,174 (88.9)	1,321 (100)
Female	63 (42.9)	552 (47)	615 (46.6)
Patient age			
[0–5]	26 (17.7)	365 (31.1)	391 (29.6)
[6–17]	29 (19.7)	259 (22.1)	288 (21.8)
[18–44]	31 (21.1)	189 (16.1)	220 (16.7)
[45–64]	30 (20.4)	162 (13.8)	192 (14.5)
[65+]	31 (21.1)	199 (17)	230 (17.4)
Race/ethnicity			
Black	80 (54.4)	465 (39.6)	545 (41.3)
Hispanic	4 (2.7)	47 (4)	51 (3.9)
Other	22 (15)	181 (15.4)	203 (15.4)
White	41 (27.9)	481 (41)	522 (39.5)
Comorbidities			
Asthma	28 (19)	113 (9.6)	141 (10.7)
Hypertension	76 (51.7)	452 (38.5)	528 (40)
Kidney disease	47 (32)	360 (30.7)	407 (30.8)
Immunosuppression	83 (56.5)	548 (46.7)	631 (47.8)
Diabetes	34 (23.1)	228 (19.4)	262 (19.8)
Heart failure	31 (21.1)	183 (15.6)	214 (16.2)
Atrial fibrillation	24 (16.3)	117 (10)	141 (10.7)
Cerebrovascular disease	29 (19.7)	211 (18)	240 (18.2)
Cancer	72 (49)	568 (48.4)	640 (48.4)
Coronary artery disease	51 (34.7)	342 (29.1)	393 (29.8)
Lung disease	92 (62.6)	537 (45.7)	629 (47.6)
Smoker	45 (30.6)	185 (15.8)	230 (17.4)
ED visit	110 (74.8)	850 (72.4)	960 (72.7)
Admitted	110 (74.8)	743 (63.3)	853 (64.6)
EV-related hospitalization	107 (72.8)	698 (59.5)	805 (60.9)
ICU-level of care	29 (19.7)	229 (19.5)	258 (19.5)
Supplemental oxygen	75 (51)	521 (44.4)	596 (45.1)
Expired	5 (3.4)	48 (4.1)	53 (4.0)

Among EV-D68-positive patients, chronic comorbid conditions were common, particularly those affecting the respiratory and cardiovascular systems. Asthma was present in 19.0% of cases, and lung disease in 62.6%. Cardiovascular comorbidities were frequently observed, including hypertension (51.7%), coronary artery disease (34.7%), heart failure (21.1%), and atrial fibrillation (16.3%). Immunosuppression was present in 56.5% of cases, and nearly half (49.0%) had a history of cancer.

Most EV-D68-positive patients required hospitalization (72.8%), and more than half required supplemental oxygen (51.0%). Intensive care unit-level care was required in 19.7% of cases. Overall mortality among EV-D68-positive patients was 3.4%.

#### Pattern of EV-D68 circulation

**Fig 1:**
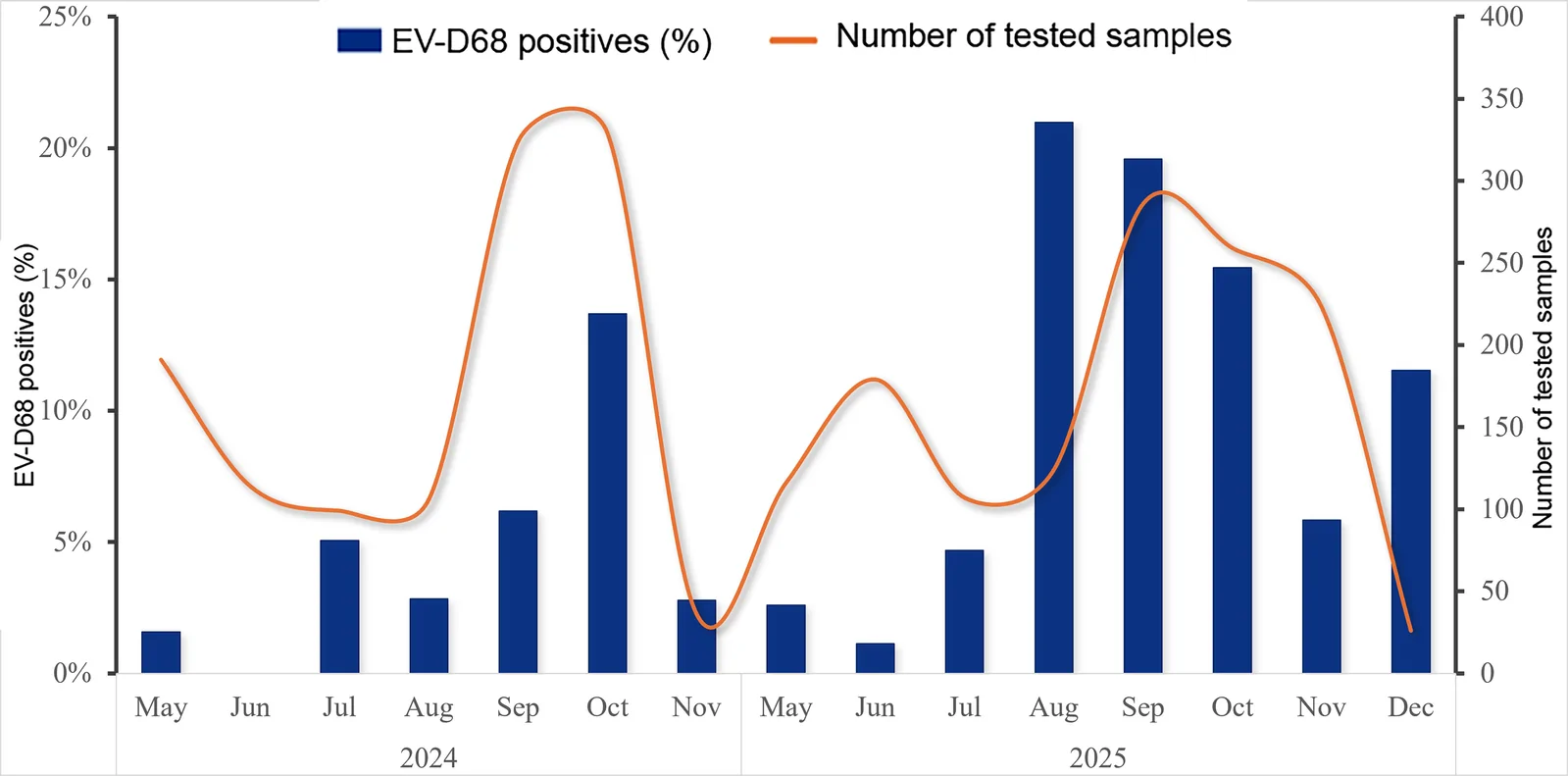
Enterovirus D68 cases identified from the Johns Hopkins Health System between 2024 and 2025.

In 2025, EV-D68 positivity remained low through May and June before increasing in July (Fig. 1). Positivity peaked in August at approximately 21%, then remained elevated in September (~20%) and October (~15%), before declining through November and December. Testing volume increased during late summer and early fall, aligning with the period of highest positivity.

Compared to 2024, the 2025 season exhibited a higher peak and a longer duration of detections. In 2024, positivity peaked in October at around 14% before declining rapidly. In contrast, 2025 saw an earlier onset, a sharper peak, and higher positivity from July through October.

### Phylogenetic analysis and recombination detection

**Fig 2:**
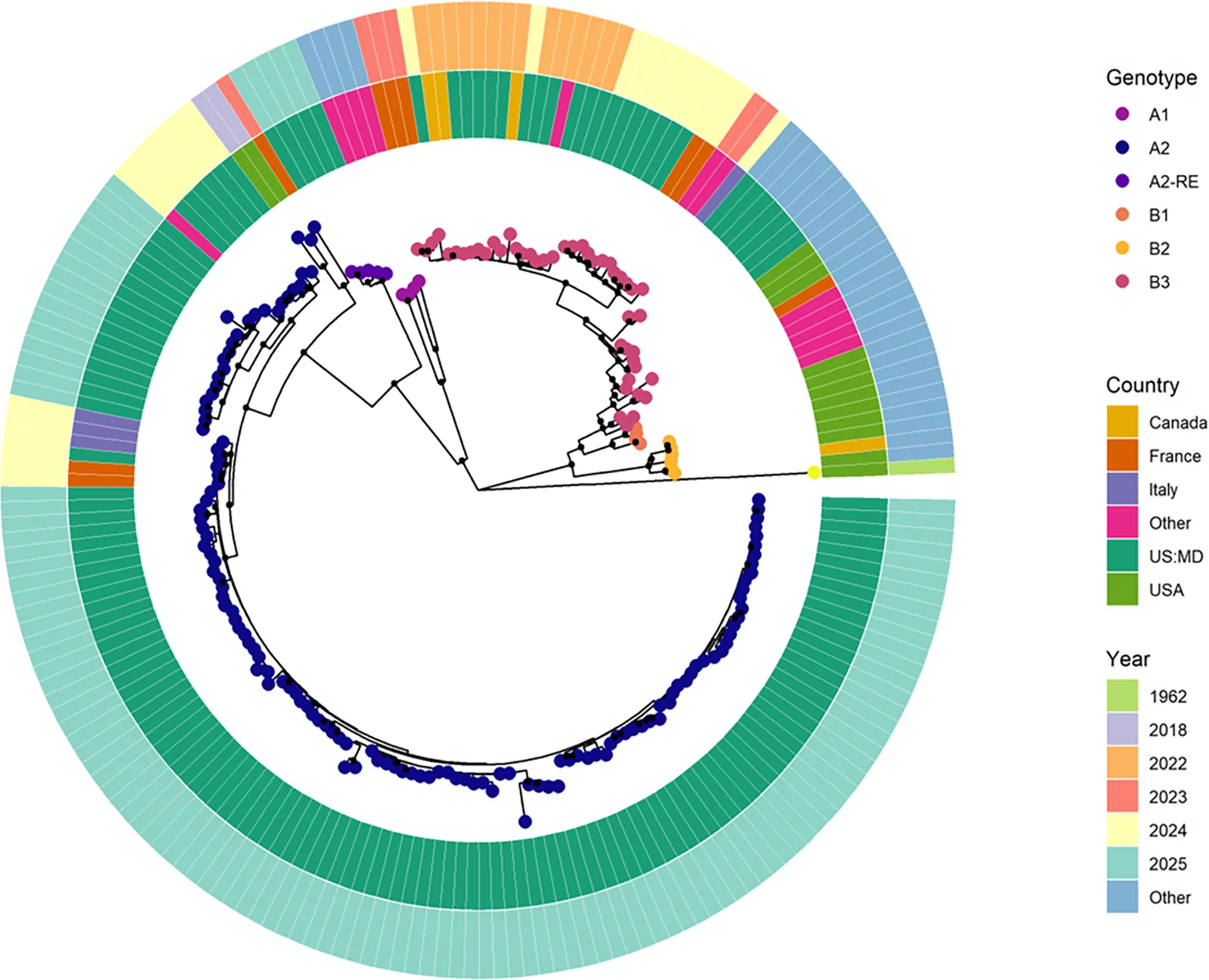
Phylogenetic relationships of EV-D68 strains identified within JHHS in 2025. Complete genome sequences from publicly available global isolates were included for contextual comparison. The phylogenetic tree was constructed using complete genome sequences and inferred by the maximum-likelihood method implemented in IQ-TREE 2, applying the TIM+F+R4 nucleotide substitution model with 1,000 bootstrap replicates. The tree was rooted using the prototype Fermon strain. Bootstrap support values ≥90% are indicated by black dots on the corresponding branches.

A total of 147 EV-D68 samples were sequenced, and complete genomes were successfully recovered from 81% (119/147). Phylogenetic analysis of complete genomes showed an exclusive circulation of the A2 subclade in Maryland during 2025, with all sequences clustering within this lineage with a bootstrap of 100 (Fig. 2). Notably, five genomes formed a distinct, monophyletic cluster that branched near A2 references. This emerging cluster was named A2-Re and exhibited limited intra-lineage genetic diversity, clustering tightly, consistent with a recent common ancestor.

**Fig 3:**
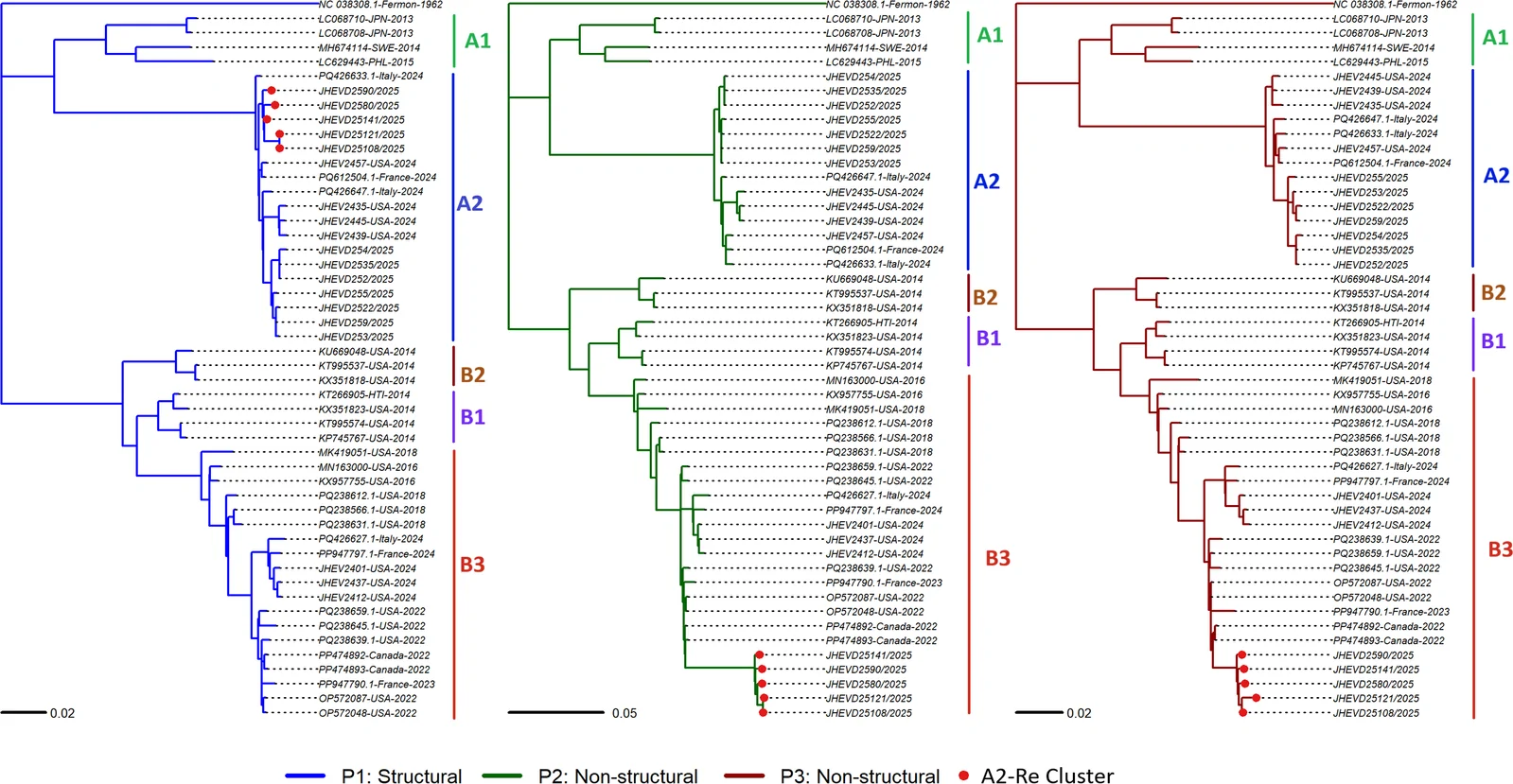
Phylogenetic analysis of the P1, P2, and P3 coding regions of EV-D68 strains from this study and globally available reference sequences. Maximum-likelihood phylogenetic trees were inferred using IQ-TREE 3 from alignments of approximately 2,586 nt (P1, blue), 1,728 nt (P2, green), and 2,256 nt (P3, red).

To investigate its evolutionary origin, we constructed subgenomic trees for the P1 (structural), P2, and P3 (non-structural) regions. We supplemented each data set with closely related sequences identified via BLAST. These analyses revealed clear topological incongruence. In the P1 tree, the cluster grouped tightly with 2024 and 2025 A2 viruses, consistent with the whole-genome phylogeny (Fig. 2). In contrast, the P2 and P3 trees placed these same genomes within the B3 subclade, forming a well-supported cluster with viruses that circulated in Canada and the United States in 2022 (Fig. 3). Within the P2 tree, the cluster exhibited longer branch lengths than typical B3 sequences, suggesting this region may be a chimera derived partly from an A2 lineage or has accumulated unique mutations. This shift in phylogenetic placement between the structural and non-structural regions indicated a recombination event, in which the P1 region was derived from an A2 parental strain, whereas part of the P2 region and the entire P3 region originated from a B3 lineage.

**Fig 4:**
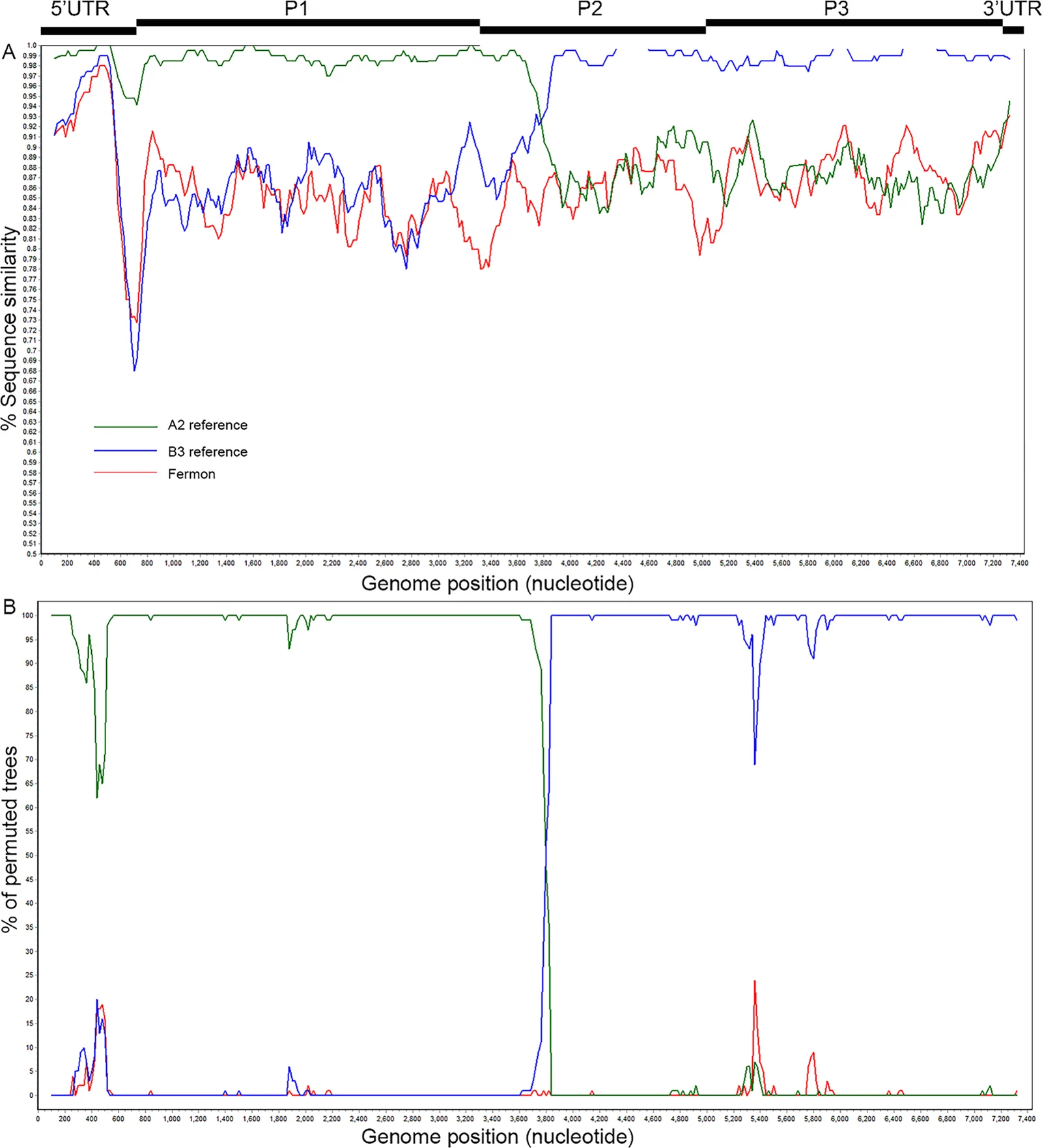
Recombination analysis of EV-D68 A2/B3 recombinant genomes using SimPlot and BootScan. Similarity (**A**) and bootstrap support (**B**) plots were generated with SimPlot v3.5.1 using a 200 bp sliding window and a 20 bp step size. The recombinant Maryland sequences (A2-Re_2025) show high similarity to A2 strains from Maryland (2025) in the P1 (structural) region and to B3 strains from Canada and Maryland (2022) in the P2–P3 (non-structural) regions, with a clear recombination breakpoint near nucleotide position 3,700.

To further confirm the recombination event and precisely locate the breakpoint, SimPlot analysis revealed a recombinant genome structure characterized by alternating regions of high sequence similarity to A2 and B3 reference strains (Fig. 4A). The plot displayed a sharp transition in similarity profiles near nucleotide position 3,700, corresponding to the 2A/2B junction.

This pattern was corroborated by BootScan analysis (Fig. 4B), which demonstrated a distinct crossover between parental lineages: the recombinant strain showed high similarity to A2 strain sequences across the P1 region and to B3 reference sequences across a part of the P2 region and the entire P3 region.

Visual inspection of the alignment using IGV confirmed uniform read coverage across the genome and no evidence of mixed populations or contamination, excluding co-infection as a potential cause for the observed mosaic signal.

**Fig 5:**
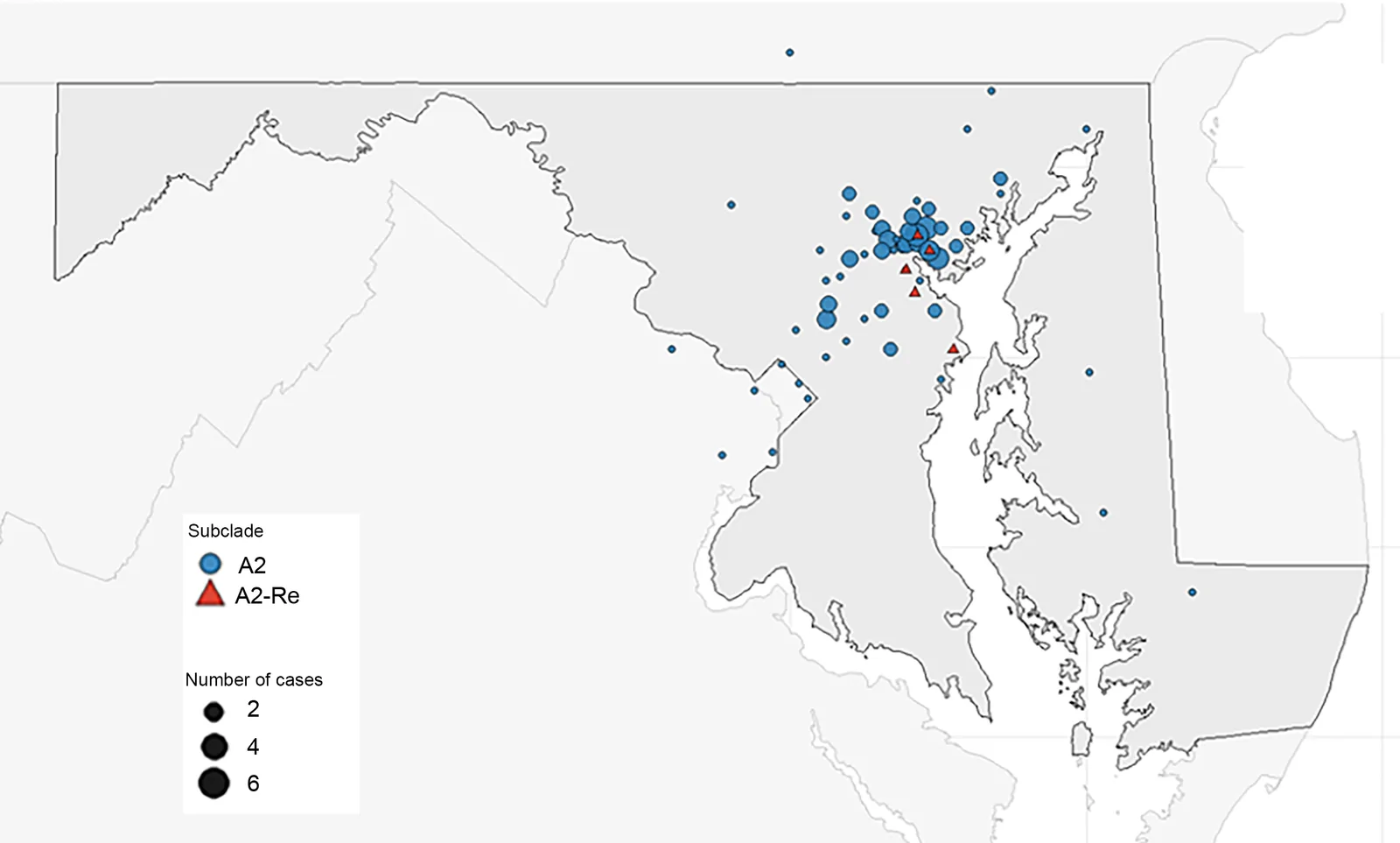
Geographic distribution of EV-D68 A2 and A2-Re cases mapped by patient ZIP code, Maryland, 2025. A2 and A2-Re cases were, respectively, represented by blue circles and red triangles. The map was generated using R.

All five patients infected with the A2-Re recombinant lineage were females. Cases were temporally clustered, with four detected in September and one in October 2025. These infections were identified across different geographic areas, suggesting local transmission (Fig. 5). Four of the five cases occurred in pediatric patients. Most individuals (4/5) required hospitalization, including two who were admitted to the intensive care unit and required supplemental oxygen.

The remaining A2 genomes segregated into two distinct subclusters. The first and predominant subcluster, comprising 99 of 114 sequences, was defined by two signature amino acid substitutions (2A: V31I and 3D: H332Y). Phylogenetically, this subcluster was most closely related to a minor A2 lineage circulating in the United States in 2024, as well as to contemporaneous genomes identified in France and Italy in 2024. It also showed close genetic relatedness to sequences detected in Sweden and France in 2023, suggesting recent international spread.

The second subcluster, comprising 15 of 114 sequences, was characterized by three signature amino acid substitutions (VP3: V153I; 2B: L38I; 3D: D49N). This lineage clustered with sequences primarily detected in the United States and China in 2024.

Notably, comparison of the partial genome region (3,700 bp) of the A2 recombinant lineage (A2-Re) with the non-recombinant A2 genomes identified in this study showed that A2-Re clustered closely with the predominant 2025 subcluster. In the shared genomic region, A2-Re sequences carried the same defining amino acid substitution (VP3: V153I), supporting a close evolutionary relationship between the recombinant and the dominant circulating A2 lineage.

## DISCUSSION

In this study, we describe an off-season surge of EV-D68 in JHHS in 2025, and the identification of a novel recombinant, A2-Re. The 2025 outbreak was characterized by increased monthly case counts compared with the 2024 season. Phylogenetic and recombination analyses provided strong evidence that A2-Re emerged from a recombination event between co-circulating contemporary A2 and B3 parental lineages, resulting in a recombinant genome with a P1 region derived from an A2 virus and the P2–P3 regions originating from a B3 virus.

Since its re-emergence in 2014, EV-D68 activity in the United States has typically followed a predictable biennial pattern, with peaks occurring in even years and concentrated in late summer and early fall (1, 10, 29, 39–41). This periodicity has been attributed to dynamic population immunity and ongoing viral evolution (42, 43). However, the 2025 outbreak suggests that this historically consistent seasonal pattern may be changing.

Notably, off-season resurgences of EV-D68 have previously been reported, particularly in Europe (44–47). Similar atypical activity was observed in the United States in 2021, although at relatively low levels (29), as well as in Senegal (48). These observations suggest that deviations from the biennial autumn pattern are not unprecedented and may reflect evolving transmission dynamics on a global scale.

Whole-genome phylogenetic analysis demonstrated exclusive circulation of the A2 subclade in Maryland during 2025, indicating a marked clade replacement relative to prior U.S. outbreaks, which were predominantly associated with B subclades. This finding aligns with recent reports describing the increasing global predominance of the A2 subclade (10, 28, 46, 49, 50).

Notably, the increased burden among older adults observed in this study, reflected by a median age of 36 years and the disproportionate representation of individuals aged ≥45 years, may be associated with the dominance of the A2 subclade. Prior studies have suggested that A2 lineages circulate more frequently among adults and older individuals compared with younger children (1, 10, 51–55).

The presence of two distinct A2 subclusters in 2025 revealed that the predominant lineage was most closely related to a minor A2 cluster circulating in the United States in 2024. Such shifts may reflect selective pressure imposed by population immunity, whereby prior exposure to dominant 2024 variants reduced susceptibility to that cluster while permitting expansion of antigenically distinct subvariants. The A2-Re cluster grouped with A2 viruses in the P1 region, and with B3 viruses from Canada and the United States (2022) in the P2–P3 regions. The SimPlot and BootScan analyses supported this topological incongruence, identifying a recombination breakpoint near the 2A/2B junction (~nt 3,700), a well-known recombination hotspot in enteroviruses (56–58). Notably, most previously reported EV-D68 recombination events have occurred in the 5′ untranslated region (5′ UTR) (25, 26, 59).

The emergence of A2-Re underscores that recombination remains an active driver of EV-D68 evolution. While previous reports of EV-D68 recombinants have been sporadic (24–27), our detection of a cluster of five nearly identical recombinant genomes in a single season from distinct geographic areas suggests local circulation.

A recent global study on the evolution and transmission dynamics of EV-D68 (28) reported limited recombination events, primarily among B3 subclade viruses from Canada, and did not confirm the more frequent recombination patterns described in earlier studies. According to that analysis, the few Canadian EV-D68 B3 sequences showing possible recombination did not exhibit clear phylogenetic discordance across the P1, P2, and P3 regions, in contrast to the findings of the present study.

The functional implications of the A2-Re recombination event could be substantial. The P1 region encodes the capsid proteins, which are the primary determinants of cell receptor binding, tissue tropism, and antigenicity. Therefore, the A2-derived P1 region suggests that this recombinant may retain the receptor usage and potentially the transmission characteristics of contemporary A2 strains. Conversely, the P2 and P3 regions encode non-structural proteins, including proteases and the RNA polymerase, which are critical for viral replication, immune evasion, and virulence (60, 61). The acquisition of a B3-derived non-structural backbone could potentially alter viral replication kinetics, pathogenicity, or host interactions.

Although the number of cases was small, the proportion requiring hospitalization, including ICU-level care, indicates that A2-Re infections were identified in patients with moderate to severe respiratory illness. The limited genetic divergence and cohesive clustering of this lineage are consistent with recent emergence and subsequent local transmission.

Several limitations should be considered when interpreting these findings. First, surveillance was conducted within a single healthcare system in Maryland, which may not fully capture the geographic diversity of circulating EV-D68 strains across the United States. Second, testing practices at our institution may have introduced selection bias. Although extended respiratory panel testing that includes RV/EV is not restricted in inpatient settings, it is primarily recommended for hospitalized or higher-risk patients (62, 63). Consequently, case detection may have been biased toward individuals with more severe illness, specific age groups, and a higher burden of comorbidities. Third, the number of A2-Re cases identified was small, limiting our ability to draw definitive conclusions regarding its transmissibility, clinical impact, or potential fitness advantage compared with contemporaneous A2 strains.

In conclusion, our genomic surveillance in 2025 identified the emergence and local circulation of a novel recombinant EV-D68 strain. This finding has several important implications. First, it demonstrates that recombination between distinct EV-D68 clades is an ongoing evolutionary process that can generate novel, viable subclades or clades. Second, it emphasizes the critical importance of whole-genome sequencing and recombination-aware phylogenetic analyses for accurate viral classification. Relying solely on partial genomic sequences (e.g., VP1) would have misclassified this cluster as a typical A2 strain. Finally, the continued genetic plasticity of EV-D68 poses a potential public health challenge, as future recombination events could theoretically generate strains with enhanced transmissibility, neurotropism, or antigenic novelty. Continued and expanded genomic surveillance is therefore essential to monitor the emergence and spread of such recombinant forms and to understand their clinical impact, particularly their association with severe neurological diseases such as AFM.

## Data Availability

The EV-D68 genome sequences generated in this study have been deposited in GenBank under accession numbers PZ210516–PZ210604.
